# Differential Proliferation Effects after Short-Term Cultivation of Mouse Spermatogonial Stem Cells on Different Feeder Layers

**DOI:** 10.22074/cellj.2019.5802

**Published:** 2019-02-25

**Authors:** Hossein Azizi, Hatef Ghasemi Hamidabadi, Thomas Skutella

**Affiliations:** 1 Faculty of Biotechnology, Amol University of Special Modern Technologies, Amol, Iran; 2 Department of Anatomy and Cell Biology, Faculty of Medicine, Mazandaran University of Medical Sciences, Sari, Iran; 3 Immunogenetic Research Center, Department of Anatomy and Cell Biology, Faculty of Medicine, Mazandaran University of Medical Sciences, Sari, Iran; 4 Institute for Anatomy and Cell Biology III, Medical Faculty, Heidelberg University, Im Neuenheimer Feld 307, 69120 Heidelberg, Germany

**Keywords:** Feeder Layers, Proliferation, Spermatogonial Stem Cells

## Abstract

**Objective:**

Spermatogonial stem cells (SSCs) provide the cellular basis for sperm production transforming the male’s genetic information to the next generation. We aimed to examine the effect of different feeder layer on proliferation of SSCs.

**Materials and Methods:**

In this experimental study, we compared the *in vitro* effects of the co-culture of mouse SSCs with mouse embryonic fibroblasts (MEFs), sandos inbred mice (SIM) embryo-derived thioguanine- and ouabain- resistant (STO) feeders, and neonate and adult testicular stroma cell (TSC) feeders on the efficiency of mouse SSC proliferation and colony formation. Cells were cultivated on top of MEFs, STO, and neonate and adult TSCs feeder layers for 30 days. The number and diameter of colonies and also the number of cells were evaluated during day 7, 15, 25, and 30 of culture. The mRNA expression of germ cells and somatic cells were analyzed.

**Results:**

In our study, we observed a significant difference in the proliferation rates and colony size of SSCs among the groups, especially for MEFs (P<0.05). SSCs can proliferate on MEFS, but not on STO, neonate or adult TSCs. Using immunocytochemistry by KI67 the proliferative activities of SSC colonies on MEFs were confirmed. The results of Fluidigm real-time polymerase chain reaction (RT-PCR) showed a high expression of the germ cell genes the promyelocytic leukemia zinc finger protein (*PLZF*), deleted in azoospermia-like (*DAZL*), octamer-binding transcription factor 4 (*OCT4*), and DEAD (Asp-Glu-Ala-Asp) box polypeptide 4 (*DDX4* or *VASA*) in SSCs, and a low expression of these genes in the feeder layers. Furthermore, we observed a higher expression of vimentin and integrin-B1 in feeder layers than in SSCs (P<0.05).

**Conclusion:**

Based on the optimal effect of MEFs for better colonization of SSCs, these feeder cells seem to be appropriate candidates for SSC cultures prior to transplantation. Therefore, it is suggested using these feeder cells for SSC cultivation.

## Introduction

The spermatogonial stem cells (SSCs) are located within a stem cell compartment in the basal part of the seminiferous tubules. The testicular tubules are encompassed by peritubular tissue, which consists of a basement membrane located between Sertoli cells of the seminiferous epithelium and myoepithelial cells within the interstitial space (1). Interstitial tissue patches with blood vessels, macrophages, and Leydig cell islands are found around the seminiferous epithelium. Differentiation and self-renewal of SSCs are partially triggered by secretory factors of these types of somatic cells (2). SSC self-renewal and spermatogonial differentiation can be regulated by extrinsic growth factors and cytokines from the somatic environment, and the molecular intrinsic genetic programs within germ cells.

Based on the current knowledge on SSCs, they can be cultivated *in vitro* with specific culture media and feeder layers, as reported in various studies (3-6). Only a few reports exist about SSCs culturing without feeders (7), as the feeder layers are known to be essential factors in SSCs cultivation (8, 9). 

At this point, various types of feeder layers are employed in SSC cultivation. Fibroblast cells produce various growth factors, including basic fibroblast growth factor-2 (FGF2) (10), transforming growth factor-ß2 (11), extracellular matrix proteins (12), activin, Wnts, and antagonists of bone morphogenetic proteins (BMPs) (13), which are important in maintenance of stem cells. It is common to utilize primary mouse embryonic fibroblast (MEF) feeders or STO feeder cells for culturing pluripotent stem cells originating from germlines such as embryonic carcinoma (EC) stem cells, embryonic stem (ES) cells, or embryonic germ (EG) cells.

Similar to the feeder supported stem cell cultures mentioned above, nowadays, several SSC studies utilized MEF feeder cells (6, 14, 15). Another well-known mouse cell line was the origin of different kinds of feeder cells, the STO feeder cells, which can substitute MEFs. On STO layers, SSCs were sustained in culture for months, as reported in a study by Nagano et al. (16). Especially, Oatley et al. (17) and Mohamadi et al. (18) used STO feeder cells for *in vitro* SSC cultivation. The proliferation of SSCs was also described to be enhanced by yolk sac-derived endothelial cell (C166) feeder layers (19). In addition, testicular feeders containing CD34-positive cells have been shown to be useful for the cultivation of GPR125 (an orphan adhesion type G-protein-coupled receptor)-positive SSCs (20).

The goal of this research was to assess the effectiveness of different culture systems (MEF, STO, and neonate and adult TSCs) for *in vitro* mouse SSC germ cell culturing. 

## Materials and Methods

### Digestion of testis

Amol University of Special Modern Technologies Ethical Committee (Amol, Iran) approved the animal experiments. Testis cells from 6 days to 6 months-old Oct4-promoter reporter GFP from C57BL/6 transgenic mouse strain were isolated after decapsulation and treatment according to a one-step enzymatic digestion protocol. After removing the tunica albuginea, dissociated testicular tissue was placed in digestion solution, which contained collagenase IV (0.5 mg/ml), DNAse (0.5mg/ ml) and Dispase (0.5 mg/ml) in HBSS (Hank’s Balanced Salt Solution) buffer with Ca^++^ and Mg^++^ (PAA, USA) at 37°C for 8 minutes. Digestion enzymes were purchased from Sigma Aldrich. The digestion enzymes were stopped with 10% ES cell-qualified fetal bovine serum (FBS, Invitrogen, USA) and then pipetted to obtain a single cell suspension. After centrifugation, the specimens were washed with DMEM/F12 (Invitrogen, USA), filtered through a 70 µm strainer and centrifuged for 10 minutes at 1500 rpm (6). 

### Preparation and culture of the different feeder cells

#### Sandos inbred mice embryo-derived thioguanine- and ouabain-resistant feeders 

STO cell line, which was originally derived by A. Bernstein, Ontario Cancer Institute, Toronto, Canada from a continuous line of SIM mouse embryonic fibroblasts, was ordered commercially from ATCC (STO (ATCC® CRL-1503™). 

For maintenance of STO feeder cells were cultured in T-75 tissue culture flask at 37°C and 5% CO_2_ in ATCC-formulated Dulbecco’s Modified Eagle’s Medium (DMEM, Invitrogen, USA) supplemented with FBS to a final concentration of 10%. The cells were routinely passaged when reaching 90% of confluency. The proliferation of STO cells was inactivated either by .-irradiation or mitomycin C (10 mg /ml) treatment.

#### Mouse testicular stromal feeder cells 

Testicular stroma cells (TSCs) were prepared both from the testis of neonate and adult mice. After digestion of the testicular tissue, the whole cell fraction was cultured in T-75 tissue culture flask at 37°C and 5% CO_2_ on culture media by serially passaging 2-3 times over the span of 2 weeks in DMEM containing 10% FBS. The feeder cells were passaged to a new culture flask when reached 90% confluency. After passage 2-3, TSCs were further treated for mitotic inactivation with mitomycin C (10 mg /ml).

### Mouse embryonic feeder cells

For the derivation of MEF cells mouse embryos from E13-E14, pregnant mice were used. After sacrifice of the pregnant females mice with CO_2_ asphyxia, the embryos were retrieved by removing the placental and fetal membranes. Afterward, the embryos were washed with Hank’s Balanced Salt Solution (HBSS) buffer, followed by excision of the intestinal from the embryos. This was followed by transferring the embryo carcasses to a new plate with HBSS buffer. The tissues were minced by aspiration through a syringe. This was followed by digestion with trypsin or collagenase-dispase (1mg/ ml) for 15-20 minutes. The digesting enzymes were inactivated with 15% serum, and the cells were pipetted several times in order to break up the remaining pieces of tissue. For maintenance, MEFs were cultured in DMEM containing 10% FBS in T-75 tissue culture flask at 37°C and 5% CO_2_. MEF cells were passaged when the culture cells reached 90% of confluence. In passage 3-4, MEF cells were used for mitotic inactivation with .-irradiation or mitomycin C treatment. 

### The culture of testicular cells 

The supernatant was removed, and the testicular cell suspension was plated onto 0.2% gelatin-coated culture dishes (approximately 0.2-0.5×10^5^ cells per 3.8 cm^2^ for neonate and 2×105 cells per 3.8 cm^2^ for adult mice) in SSCs medium, which consisted of StemPro-34 medium, 1% N2-supplement (Invitrogen, USA), 6 mg/ml D+glucose (Sigma Aldrich, USA), 5 µg/ml bovine serum albumin (Sigma Aldrich, USA), 1% L-glutamine (PAA, USA), 0,1% ß-mercaptoethanol (Invitrogen, USA), 1% penicillin/streptomycin (PAA, USA), 1% MEM vitamins (PAA, USA), 1% non-essential amino acids (PAA, USA), 30 ng/ml estradiol (Sigma Aldrich, USA), 60 ng/ml progesterone (Sigma Aldrich, USA), 20 ng/ml epidermal growth factor (EGF, Sigma Aldrich, USA), 10 ng/ml FGF (Sigma Aldrich, USA), 8 ng/ml GDNF (Sigma Aldrich, USA), 100 U/ml human leukemia inhibitory factor (LIF, Millipore, USA), 1% ES cell qualified FBS, 100 µg/ml ascorbic acid (Sigma Aldrich, USA), 30 µg/ml pyruvic acid (Sigma Aldrich, USA) and 1 µl/ml DL-lactic acid (Sigma Aldrich, USA) at 37°C and 5% CO_2_ in air. The molecular and functional characterization of SSCs were established similarly as described in our previous study (6). In the next step, for analyzing the efficiency of mouse SSCs growth and colony formation, about 4000 SSCs were plated on a 24well plate, in which each well was coated with MEFs from C57BL/6 (C57-MEF), MEFs from CF1 mouse (CF1MEF), STO, neonate testicular stromal cells (N-TSCs), and adult TSCs (A-TSCs) feeder layers. Afterward, the number and diameter of the colonies, as well as the number of cells were evaluated during day 7, 15, 25, and 30 of culture. The diameter of colonies was measured by the ImageJ software. For the measurement of the number of cells, as we mentioned above, we plated 4000 cells in each well of 24 well plates, and after trypsinization, cells were counted during day 7, 15, 25, and 30. 

### Gene expression analyses on the Fluidigm Biomark system

Dynamic array chips were employed to measure the expression of the genes by a Fluidigm Real-time polymerase chain reaction (PCR) system (6). All Taqman real-time PCR assays were provided by Thermo Fisher Scientific, for octamer-binding transcription factor 4 (*OCT4*) the assay Mm03053917_g1, deleted in azoospermia-like (*DAZL*) Mm00515630_m1, *VASA* Mm00802445_m1, INTEGRIN-B1 Mm01200043_m1, zinc finger and BTB domain containing 16 (*PLZF*) Mm01176868_m1, VIMENTIN Mm00619195_g1, G-protein coupled receptor 125 (GPR125), Tetraspanin-29 (*CD9*) Mm00514275_g1, and the housekeeping gene glyceraldehyde-3-phosphate dehydrogenase (GAPDH) Mm99999915_g1, which was used for normalization of the different types of cultured cells. The cultured cells included neonate SSCs (N-SSCs), adult SSCs (A-SSCs), C57-MEF, CF1-MEF, STO, N-TSCs, and A-TSCs. In each sample, about 50 cells were manually selected from the cultures with a micromanipulator, lysed with special lysis buffer containing 9 µl RT-PreAmp Master Mix (5.0 µl Cells Direct 2× Reaction Mix (Invitrogen, USA), 2.5 µl 0.2× assay pool, 0.2 µl RT/Taq Superscript III (Invitrogen, USA), and 1.3 µl TE (Tris-EDTA, Invitrogen, USA) buffer and immediately frozen and stored at -80°C. The number of targeted transcripts was quantified using TaqMan real-time PCR on the BioMark real-time quantitative PCR (qPCR) system (Fluidigm). Every sample was examined in two technical replicates. The Ct values achieved by the BioMark System were analyzed by GenEx software from the MultiD analysis (6). 

### Immunocytochemical staining

Cells were cultured in 24 well plates and fixed with 4% paraformaldehyde. After rinsing with phosphate buffered solutions (PBS, Invitrogen, USA) the samples were permeabilized with 0.1% Triton (Invitrogen, USA)/PBS and blocked with 1% bovine serum albumin (BSA, Sigma Aldrich)/PBS. After removing the blocking solution, the cellswere incubated overnight with the primary Ki67 antibody(Sigma Aldrich, USA). After rinsing, the process was followed by incubation with species-specific secondary antibodies, which were conjugated with fluorochrome; the labeledcells were counterstained with 0.2 µg/ml 4’, 6-diamidino2- phenylindole (DAPI, DAPI, Sigma Aldrich, USA) for 3minutes at room temperature and fixed with Mowiol 4-88 reagent (Merck, USA). Labeled cells were examined with aconfocal microscope Zeiss LSM 700, and images were taken with a Zeiss LSM-TPMT camera (6). 

### Statistical analysis

The experiments were replicated at least 3 times. The average for gene expressions in groups was calculated, and the groups were evaluated using one-way analysis of variance (ANOVA) followed by the Tukey’s post-hoc tests. The expression of genes was compared with non-parametric Mann-Whitney’s test. The variation between groups was considered statistically significant if a value of P<0.05 was obtained. 

## Results

**Fig.1:**
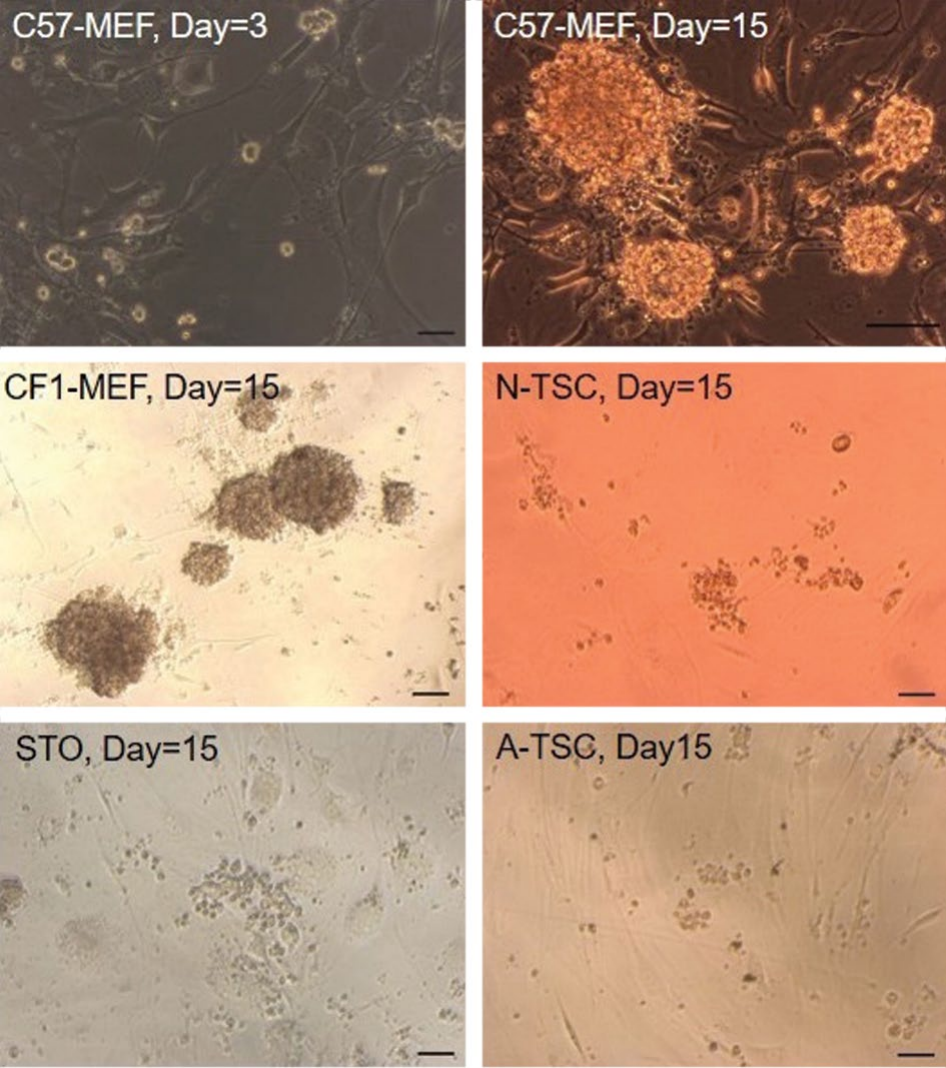
Microscopic observation of SSCs on the different feeder layer. Cultivationof SSCs on C57-MEF (MEF cells isolated from C57BL/6 mouse), CF1-MEF (MEFcells isolated from CF-1 mouse), STO (STO feeder), N-TSCs (TSCs feeder cellsisolated from neonate mouse), and A-TSCs (TSCs feeder cells isolated fromadult mouse) feeder layers. On day 15 the growth of SSCs was observed onC57-MEF and CF1-MEF feeder layer (scale bar: 100 µm). SSC; Spermatogonial stem cells, MEF; Mouse embryonic fibroblasts, STO; Sandos inbred mice embryo-derived thioguanine- and ouabain-resistant feeder, and TSC; Testicular stromal cells.

For analyzing the growth efficiency of mouse SSC on different feeder cells, SSCs were cultivated on C57-MEF, CF1-MEF, STO, N-TSCs, and A-TSCs feeder cover plates. Over time, the microscopic analysis demonstrated that the growth behavior of SSCs on C57-MEF and CF1-MEF was much stronger than on STO, N-TSCs and A-TSCs. A decrease in the number of SSCs growing on STO, N-TSCs, and A-TSCs was observed about 7 days after the initiation of the culture (Fig .1). 

**Fig.2:**
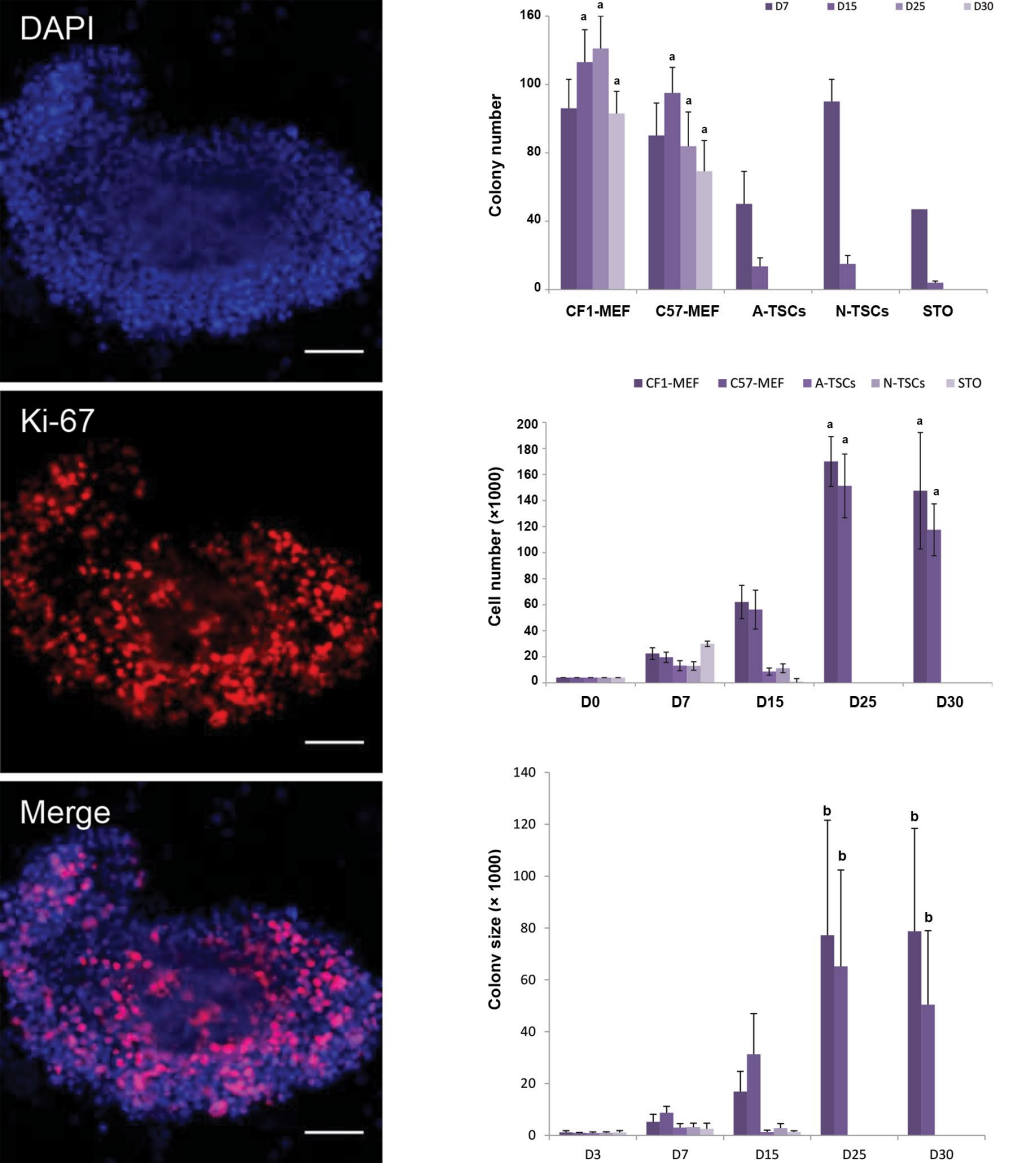
The growth analysis of SSCs on different feeder layer and immunofluorescent staining for Ki67. On C57-MEF (MEF cells isolated from C57BL/6 mouse) and CF1-MEF (MEF cells isolated from CF-1 mouse), feeder layer the number of SSCs, colonies size and colony number were significantly higher in comparison to the other types of feeder cells (P<0.05). a, b; P<0.05 in comparison to other feeder cell groups on the same day. The X-axis shows feeder cells and day. SSCs on MEF feeder layer express Ki67 protein (scale bar: 50 µm). SSC; Spermatogonial stem cells, MEF; Mouse embryonic fibroblasts, STO; Sandos inbred mice embryo-derived thioguanine- and ouabain-resistant feeder, and TSC; Testicular stromal cells.

After the transfer of SSCs onto feeders and during the initial phase of the SSC culture, under all conditions, we observed comparable growth behavior and colony formation of SSCs until about day 7. After about 7 days of the initiation of the culture, we observed reduced growing of SSC on STO, NTSC, and ATSC feeder layers, while on C57-MEF and CF1-MEF cells the SSCs continued to proliferate in number and an increase in diameter of colonies and number of SSCs colonies was observed. It should be mentioned that we did not visualize any significant difference between C57-MEF and CF1-MEF feeder layer groups. The changes in SSC number, diameter, and the number of colonies were observed to be significantly higher on days 15 and 25 compared to other time points (P<0.05). Apparently, the maximal growth of SSCs occurred by 25 days after plating the cells on MEF feeders (Fig .2), and the supportive effect of the MEF feeders seemed to diminish after day 25. 

Immunofluorescent staining showed that SSC colonies cultured on MEF feeders were strongly positive for the proliferation marker Ki67 in contrast to STO, neonate, and adult TSCs feeder layers (Fig .2). Ki67, a nonhistone nuclear protein, is expressed in the course of cell proliferation (21). 

**Fig.3:**
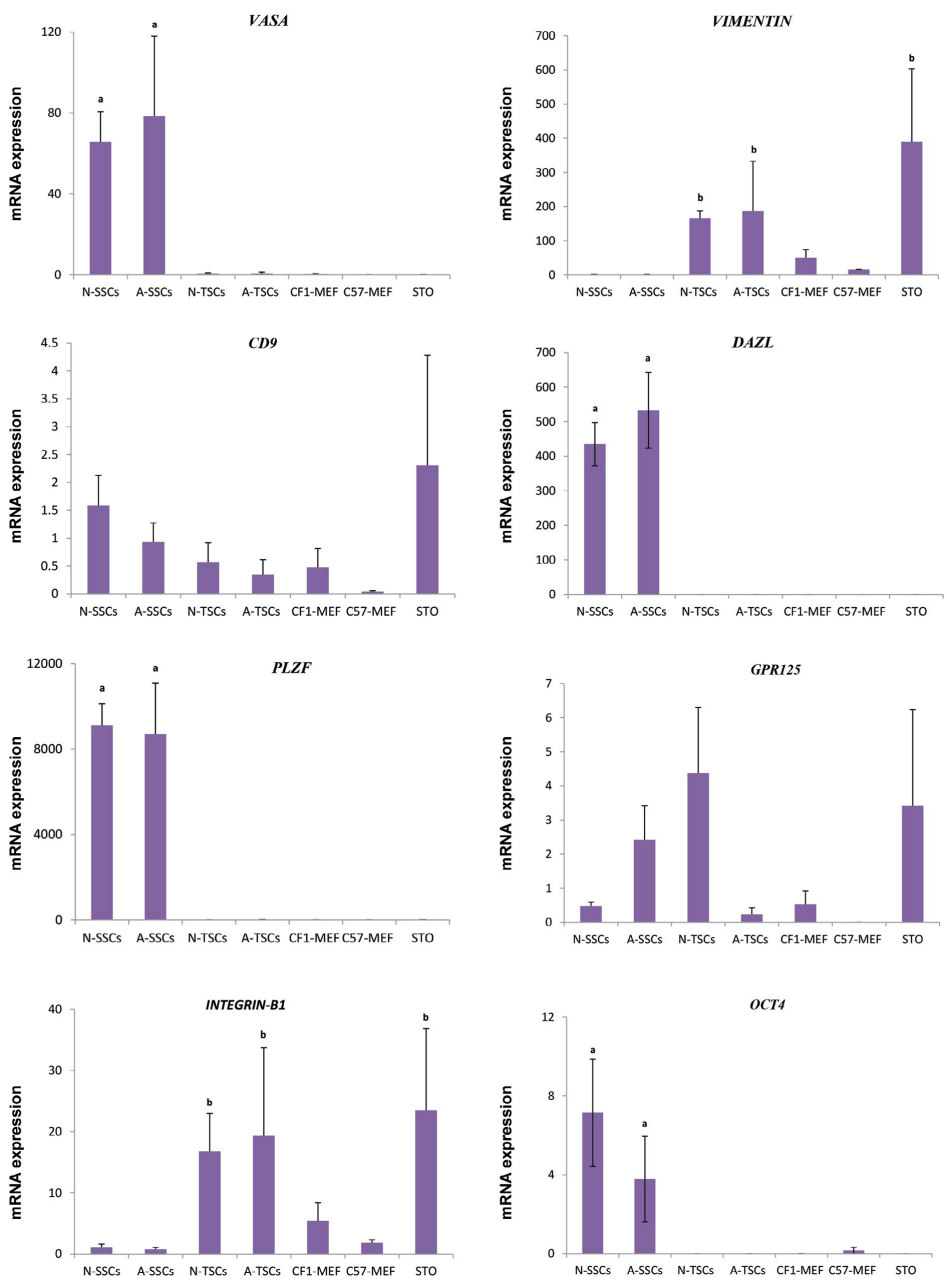
mRNA expression of germ and somatic cell markers in SSCs and feeder cells. The analysis was performed between SSCs and feeders. The significance of the difference between different groups was determined by non-parametric Mann-Whitney’s test. a, b; P<0.05 vs. other feeder cell groups. The X-axis shows feeder cells. The expression of *VASA, DAZL, PLZF,* and *OCT4* in SSCs were significantly (P<0.05) higher than the other groups. The expression of *VIMENTIN* and *INTEGRIN-B1* was significantly higher (P<0.05) in the somatic cells than in SSCs but not *CD9* and *GPR125*. SSC; Spermatogonial stem cells, MEF; Mouse embryonic fibroblasts, STO; Sandos inbred mice embryo-derived thioguanine- and ouabain-resistant feeder, and TSC; Testicular stromal cells.

To evaluate the expression of germ and somatic cell markers in SSCs and feeder cells, we analyzed the mRNA expression with Fluidigm expression profiling and Taqman assays of the following genes *PLZF, OCT4, VASA, VIMENTIN, DAZL, CD9, GPR125,* and *INTEGRIN-B1* on neonate and adult SSCs, and on feeder layers C57-MEF, CF1-MEF, STO, NTSCs, and ATSCs. We observed that the expression of *VASA, DAZL, PLZF,* and *OCT4* in N-SSCs and A-SSCs was significantly higher than in somatic cells (P<0.05). In our analysis, we observed a significantly higher expression of *VIMENTIN* and *INTEGRIN-B1* in somatic cells than N-SSCs and A-SSCs, but not for *CD9* and *GPR125* (P<0.05, Fig .3). 

## Discussion

Similar to other adult stem cells, the SSCs pass through several self-renewal and differentiation stages. During proliferation and differentiation, the extrinsic factors originating in the basal and luminal cell niches of the testicular tubules and the intrinsic gene expression pattern influence these processes (22-25). During *in vitro* cultivation, feeder layers should mimic these *in vivo* stem cell niche and might play a crucial role in self-renewal, expansion, and differentiation of SSCs by producing different soluble growth factors and contact-mediated substrates (26). Although the extrinsic factors secreted by feeder layers are only partially known, different feeder layers might cause diverse effects on self-renewal and differentiation of SSCs during cultivation. 

In this study, we reported the short-term effect of embryonic and somatic feeder layers on mouse SSC cultivation. SSCs were co-cultured on C57-MEF, CF1MEF, STO, N-TSCs, and A-TSCs feeder layers for 30 days. Our study demonstrated that the increase in the number of SSCs, the diameter, and the number of SSC colonies on MEF feeder layers was significantly higher than on STO and testicular somatic cells. 

We observed by Fluidigm real-time PCR that the expression of the germ cells genes *VASA, DAZL, PLZF,* and *OCT4* were higher in SSCs than in somatic feeder cells, while the expression of *VIMENTIN* and *INTEGRIN-B1* was higher in somatic cells in comparison to SSCs. It has been demonstrated that CD9 and GPR125 are expressed in germ cells (27), but our data also showed that the expression of these markers in somatic cells. Similarly, Shinohara et al. demonstrated that INTEGRIN-B1 is a surface marker located on SSCs (28) while we observed increased expression of INTEGRIN-B1 in somatic cells. Therefore, it seems that CD9, GPR125, and INTEGRIN-B1 cannot be regarded as specific markers for the identification of SSCs. Our observations are also supported by the data from the Human Protein Atlas (www.proteinatlas.org) which shows that these proteins are also present in somatic cells of the testis.

Similar to our findings, several other groups used MEF feeders for the long-term proliferation of SSCs in culture (6, 14, 29). We proved that somatic TSCs and STO feeder cells could not, or only to a limited degree, support SSC cultures, while several reports demonstrated the beneficial influence of these feeders on the SSC culture (19, 30-33). These various results for the cultivation of SSCs might be caused by differences in species, mouse strains used, and also different populations of SSCs in testis, which all may show different phenotypic characteristics under different culture conditions. The same reasoning can be applied to the different sources of feeder cells used for SSC co-culturing.

In conditions of the short-term culturing, the capability of STO feeders to sustain mouse neonate Thy-1 positive SSCs and bovine testicular germ cells has been reported (34, 35). In contrast to mice, in vitro cultivation and the amount of SSCs could be diminished by TM4 or SF7 somatic Sertoli cell lines (36). 

The mouse strain from which the harvested feeder cells originated from is another critical factor in SSC cultivation. DBA/2 mice produce SSCs which are unproblematic in proliferation with GDNF alone. However, different mouse strains such as C57BL/6 or 129/SvCP produce SSCs that are dependent on the soluble GDNF family receptor alpha 1 (GFRa1) and basic FGF (bFGF or FGF2) to proliferate steadily *in vitro* (6). Kanatsu-Shinohara et al. (14) have already detected the beneficial growth patterns of DBA/2-derived SSCs. According to Sariola et al. (37), a multicomponent receptor complex including RET receptor tyrosine kinase and a glycosyl phosphatidylinositol-anchored ligand-binding subunit, termed GFRa1, trigger the cellular responses to GDNF. In the majority of mouse strains, in vitro proliferation of SSCs critically depends on the addition of soluble GFRa1, since the downstream signaling is supported by RET stimulation with soluble GFRa1 (38). 

In contrast, STO feeders express the insulin- like growth factor binding protein 4 and the growth factor pigment epithelium-derived factor (39). Their various expression of growth factors may explain the greater effect of MEFs on the proliferation and colony formation of SSCs.

Further transcriptomic and proteomic analysis should aim to identify the membrane-bound and secreted molecules by MEFs facilitating the proliferation of mouse SSCs in culture. The identification of these molecules might lead to the development of a more robust culture system for SSC proliferation. A similar approach would be of tremendous advantage for the improvement of short- and long-term culturing of human SSCs. 

## Conclusion

Our data showed that the markers *VASA, DAZL, PLZF,* and *OCT4* are specific for the characterization of SSCs, but *CD9, GPR125,* and *INTEGRIN-B1* are also expressed in STO and TSCs somatic cells. Therefore, CD9, GPR125, and INTEGRIN-B1 markers are not unique for SSC identification. While some reports showed that SSCs could be cultivated and expanded on STO and somatic testicular feeder, our data showed that STO and TSC feeder could not be an ideal feeder layer for the short-term cultivation of SSCs. Our findings indicate that in comparison to STO, neonate, and adult TSC feeders, MEF feeder cells are able to better enhance SSC proliferation and expansion in the short-term cultures. In the future, it would be interesting to identify the contact-mediated substrates and soluble growth factors produced by MEF feeder cells which might be beneficial for self-renewal and expansion of mouse SSCs in short-term cultures.
